# A scoping review of the Stanford Integrated Psychosocial Assessment for Transplantation (SIPAT) for use with liver transplant candidates

**DOI:** 10.1186/s13030-026-00352-4

**Published:** 2026-02-07

**Authors:** Alberto Olivero, Marco Miniotti, Alessandro Godono, Paolo Leombruni

**Affiliations:** 1 “Rita Levi Montalcini” Department of Neuroscience, University of Turin, Turin, Italy; 2 Department of Public Health and Pediatrics, University of Turin, Turin, Italy

**Keywords:** Liver transplantation, SIPAT, Psychosocial assessment, Review, Alcohol-associated liver disease

## Abstract

**Background:**

Liver transplantation (LT) is a life-saving treatment for end-stage liver disease, and psychosocial factors significantly influence eligibility, adherence, and outcomes. The Stanford Integrated Psychosocial Assessment for Transplantation (SIPAT) was developed to standardize psychosocial evaluation across four domains: treatment readiness, social support, psychopathology, and substance use. Despite its growing global adoption, evidence regarding its validity, predictive value, and applicability, specifically in LT, remains fragmented and heterogeneous. This scoping review maps and summarizes the available empirical evidence on SIPAT use in adult LT candidates. It focuses on (1) score distributions (2), psychosocial differences by disease etiology, and (3) associations with listing decisions, post-transplant outcomes, relapse risk, and quality of life.

**Main body:**

A systematic search of PubMed, Scopus, and PsycInfo (January 2012–May 2025) identified ten eligible empirical studies, primarily observational in design, with sample sizes ranging from 22 to 2,825 participants. Across studies, a substantial proportion of LT candidates were classified as having moderate-to-high psychosocial risk, frequently exceeding a SIPAT threshold of ≥ 21, although cutoff values varied across studies. Evidence from a limited number of investigations suggests that domains related to treatment readiness and lifestyle/substance use may be particularly affected in LT populations. Studies examining etiological subgroups reported higher SIPAT scores among candidates with alcohol-associated liver disease (ALD) or acute alcoholic hepatitis compared with non-ALD candidates, although findings were not uniform across all studies. Higher total SIPAT scores were associated in several cohorts with lower likelihood of listing and with adverse post-transplant outcomes, including nonadherence and acute rejection. In ALD samples, elevated SIPAT scores were associated with increased risk of harmful alcohol relapse, independent of abstinence duration. Notably, none of the included studies evaluated associations between SIPAT scores and post-transplant quality-of-life outcomes.

**Conclusion:**

Current evidence suggests that the SIPAT is a useful framework for structuring psychosocial assessment in liver transplantation and for identifying potentially modifiable vulnerabilities relevant to listing decisions and selected post-transplant outcomes. However, substantial methodological heterogeneity, inconsistent cutoff thresholds, and limited evidence across some outcome domains, particularly quality of life, constrain comparability and clinical generalizability. Future multicenter, prospective studies are needed to harmonize cutoff values, examine sensitivity to change, and clarify the role of SIPAT in predicting long-term patient-centered outcomes.

## Introduction

Liver transplantation (LT) is a life-saving intervention for individuals with end-stage liver disease and selected hepatocellular carcinomas; however, the chronic global shortage of donor organs necessitates highly selective listing criteria to ensure optimal graft utility and long-term benefit [1]. While biomedical parameters remain central, structured psychosocial evaluation has gained increasing prominence as a critical component of candidate assessment. Current guidelines emphasize the integration of psychosocial data to guide listing and optimize post-transplant adherence, long-term survival, and health-related quality of life [2, 3].

Psychosocial risk factors—including substance use relapse, medication nonadherence, poor emotional adjustment, and lack of support—are consistently associated with adverse transplant outcomes [2, 4, 5]. Nonetheless, many transplant programs continue to rely on unstructured interviews and subjective clinical impressions, limiting reproducibility and equity in access to transplantation [6, 7].

To address this gap, the Stanford Integrated Psychosocial Assessment for Transplantation (SIPAT) was developed as a standardized tool for the assessment of four core domains: (a) Patient Readiness Level and Illness Management (“readiness”); (b) Social Support System (“social support”); (c) Psychological Stability and Psychopathology (“psychopathology”); and (d) Lifestyle and Effect of Substance Use (“lifestyle/substance use”) [8, 9]. The SIPAT generates a composite score to stratify candidates into low-, moderate-, or high-risk categories, facilitating more objective decision-making.

Since its original development, the SIPAT has been translated and validated in multiple languages—including Spanish, Italian, Japanese, Thai, and Persian—and has demonstrated satisfactory internal consistency, inter-rater reliability, and convergent validity [7, 10–13].

In liver transplantation specifically, the SIPAT has been examined in relation to candidate characteristics, listing outcomes, and selected post-transplant endpoints; however, the available evidence remains heterogeneous in design, outcome definitions, and cut-off thresholds, which limits comparability across studies.

In the LT context, SIPAT scores have been reported to vary by disease etiology (particularly alcohol-associated liver disease [ALD]), listing status, and post-transplant outcomes. Studies indicate that higher SIPAT scores predict increased likelihood of listing denial, poor adherence to immunosuppressive therapy, relapse into alcohol use, and diminished psychological adjustment post-operatively [14–17].

The need for rigorous psychosocial assessment is underscored by the shifting epidemiology of liver disease: ALD and metabolic dysfunction-associated steatotic liver disease (MASLD) are now the leading causes of liver failure in high-income countries, replacing hepatitis C [1]. These etiologies may be associated with increased psychosocial vulnerability, particularly substance use–related risk in ALD [18].

In parallel, growing attention is being paid to quality of life (QoL) after LT. Although health-related QoL (HRQoL) improves substantially post-transplant, many recipients experience persistent symptoms—fatigue, anxiety, impaired role functioning—and only a minority return to work [6]. Such outcomes are increasingly recognized as being influenced not only by medical variables but also by psychosocial resilience and support systems, domains that the SIPAT aims to systematically assess prior to transplant listing.

This scoping review aims to map and summarize the current evidence on the use of the SIPAT for adult liver transplant candidates, with three primary objectives:

1. To describe SIPAT total and subscale score distributions in LT populations.
2. To examine psychosocial profile differences by disease etiology.
3. To evaluate the SIPAT’s predictive validity regarding listing decisions, post-transplant outcomes, relapse risk, and quality of life.

## Methods

### Review design and approach

**Fig. 1:**
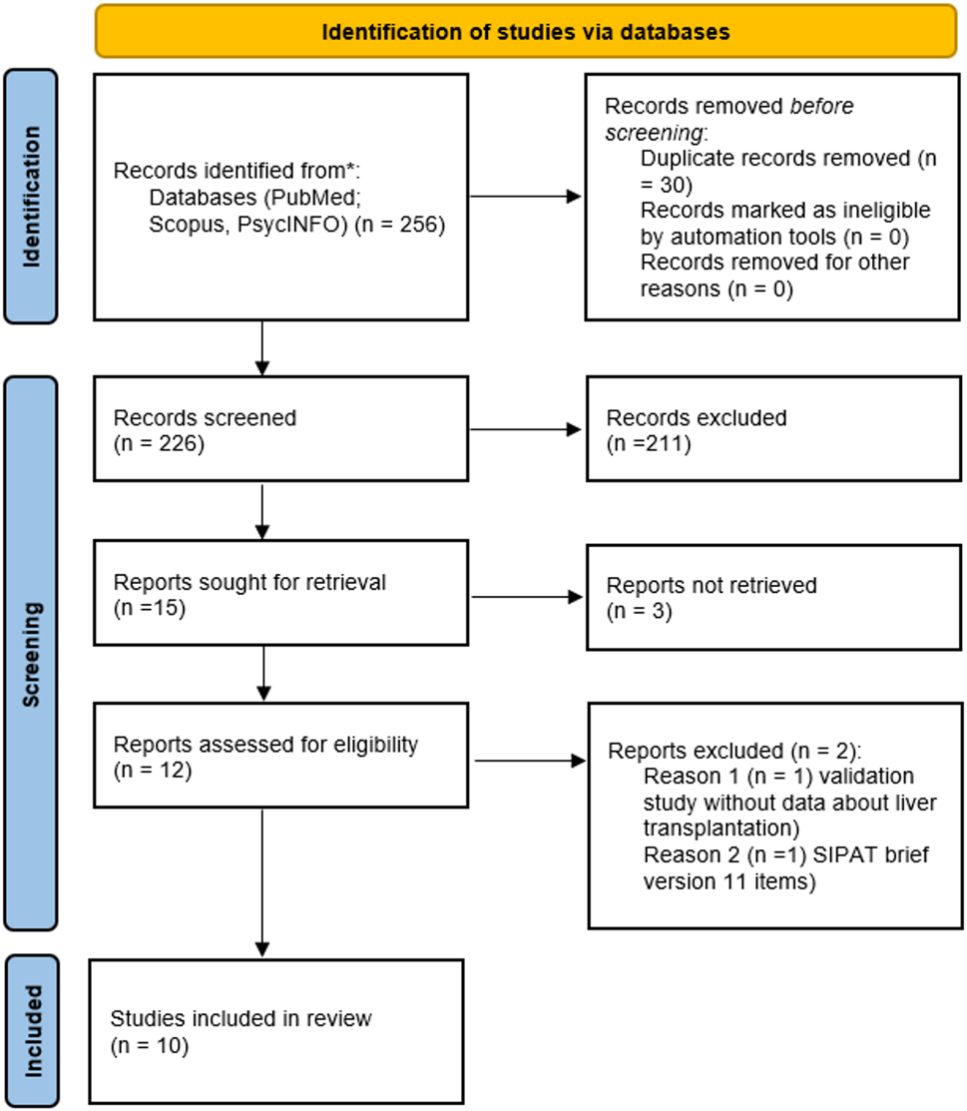
PRISMA 2020 flow diagram of study selection

This scoping review was designed to critically synthesize the literature on the use of the Stanford Integrated Psychosocial Assessment for Transplantation (SIPAT) in adult liver transplantation (LT). The methodology drew on key principles from PRISMA 2020 to ensure clarity and transparency in the search, screening, and selection process. The study selection process is presented as a PRISMA 2020 flow diagram (Fig. 1).

### Search strategy

We conducted a comprehensive search of three major biomedical databases—PubMed, Scopus, and PsycInfo—covering the period from January 2012 to May 2025. The search combined the following terms:

(“SIPAT” OR “Stanford Integrated Psychosocial Assessment for Transplantation”) AND (“liver transplantation” OR “hepatic transplant”) AND (“psychosocial assessment” OR “psychosocial evaluation”).

This search returned 256 records. Search results were exported and managed using a reference manager (Mendeley) to ensure accurate deduplication and tracking of eligibility. After removing 30 duplicates, 226 titles and abstracts were screened. Of these, 211 were excluded based on relevance.

### Study selection

We retrieved the full text of 15 potentially relevant articles. Three could not be accessed despite repeated attempts. Of the 12 articles reviewed in full, two were excluded: one a validation study unrelated to liver transplant populations and the other used a short-form SIPAT (11-item version) that was not comparable to the original instrument. As a result, 10 studies were included in the final synthesis. The full selection process is illustrated in Fig. 1.

### Inclusion and exclusion criteria

Studies that met the following criteria were included:

- Population: Adults (≥ 18 years) who had undergone psychosocial evaluation for liver transplantation and/or studies reporting post-transplant outcomes in cohorts where SIPAT was administered in the context of transplant candidacy assessment.
- Instrument: Use of the full (standard) version of the SIPAT.
- Outcomes: Reporting of SIPAT scores (total and/or subscales) and associations with clinical or psychosocial outcomes.
- Design: Observational studies (cross-sectional, prospective, or retrospective) published in peer-reviewed journals.
- Language: Full-text available in English.

Studies were excluded if they:

- Focused on pediatric or non-LT transplant populations without specific liver data.
- Presented only conceptual, theoretical, or validation work without empirical data.
- Used a modified version of the SIPAT.
- Were inaccessible in full text.

### Measure overview: SIPAT

The Stanford Integrated Psychosocial Assessment for Transplantation (SIPAT) is an 18-item clinician-rated instrument designed to structure pre-transplant psychosocial evaluation across four domains. Higher total scores indicate greater psychosocial risk. The SIPAT yields a total score and domain scores and provides an interpretative risk stratification framework [8, 9].

Given the heterogeneity in cut-off thresholds adopted across liver transplantation studies, we did not impose a single threshold a priori; instead, we extracted and reported the cut-offs and risk categories as used by each included study.

### Quality assessment

Each included study was appraised using Joanna Briggs Institute Critical Appraisal Tools (JBI-CAT) selected according to study design. Two reviewers independently assessed methodological rigor across domains such as clarity of inclusion criteria, measurement reliability, control of confounders, and appropriateness of analysis. Disagreements were resolved through discussion or consultation with a third reviewer. Risk of bias across studies was summarized narratively, and quality ratings informed interpretation but not inclusion decisions. Specifically, findings from moderate-quality studies were interpreted more cautiously and were primarily used to identify emerging patterns and evidence gaps, whereas greater weight was given to consistent findings from high-quality studies.

### Data extraction and synthesis

Two reviewers independently extracted data into a structured template, collecting the following for each study: authorship, year, country, study design, sample size, participant characteristics, SIPAT scores (mean ± SD or median [IQR]), and main clinical outcomes. In cases of disagreement, consensus was reached through discussion.

Due to substantial heterogeneity in designs, outcome definitions, and reporting formats, we performed a narrative synthesis. In line with scoping review methodology, our synthesis aimed to map the breadth of existing evidence and highlight research gaps rather than derive definitive pooled estimates.

## Results

### Overview of included studies

Ten studies met the inclusion criteria and were included in this review [14–17, 19–24]. Based on the Joanna Briggs Institute Critical Appraisal Tools, seven studies were rated as high quality and three as moderate. Key study characteristics and outcomes are summarized in Table 1.

### SIPAT Score distribution and prevalence of psychosocial risk in liver transplant candidates (*n* = 3 studies)

Multiple studies have highlighted a substantial prevalence of elevated psychosocial risk among LT candidates assessed with the SIPAT (Table 1). Zanatta et al. (2024) [24] reported that 57% of 134 candidates scored ≥ 21, with a mean score of 23.93 (SD = 12.99), particularly elevated in the “readiness/illness management” and “lifestyle/substance use” domains. Similarly, Deutsch-Link et al. (2021) [20], in a U.S. cohort of 1,357 patients, found that approximately one-third scored within the SIPAT ≥ 21 range, classifying them as “minimally acceptable” or “high-risk” candidates. In a Japanese sample, Takano et al. (2023) [22] observed a mean SIPAT score of 22.11 (SD = 8.80) among 71 LT candidates, indicating a higher psychosocial burden compared to candidates for other types of organ transplants.

Although total score distributions were reported in these cohorts, reporting of SIPAT domain/subscale score distribution was inconsistent across studies and could not be systematically summarized beyond the studies that explicitly provided domain-level findings [24].

### Psychosocial differences by disease etiology (*n* = 2 studies)

Two studies explicitly examined SIPAT differences by liver disease etiology (Table 1). Matthews et al. (2023) [17], in a cohort of 187 transplant candidates, found that ALD patients had significantly higher SIPAT scores than non-ALD patients (39.4 vs. 22.5; *p* < 0.001). Zanatta et al. (2024) [24] similarly reported higher SIPAT scores among candidates with alcoholic etiology compared with non-alcoholic etiologies (29.66 vs. 18.21). Given the limited number of studies directly assessing etiological subgroups, these findings should be interpreted as evidence mapping rather than as definitive cross-cohort generalizations.

### Association between SIPAT scores and transplant listing (*n* = 4 studies)

Across four studies evaluating listing-related outcomes (Table 1), higher SIPAT scores (or higher risk categories) were associated with lower likelihood of being listed or with listing exclusion. In a large-scale study by Sun et al. (2024) [19], among 2,762 evaluated patients, the median SIPAT score was 15 (IQR: 8–23), significantly lower among those listed for transplant (14; IQR: 8–21) compared to those not listed (17; IQR: 10–26; *p* < 0.001). Deutsch-Link et al. (2021) [20] reported that SIPAT scores ≥ 21 were independently associated with an increased likelihood of exclusion from the transplant list (adjusted OR = 1.78), with contributions from all assessed domains.

Additional evidence was provided by Zanowski et al. (2022) [15], who studied a sample of 83 patients with severe alcoholic hepatitis. In this cohort, median SIPAT scores were significantly higher among those excluded from the list (57 vs. 40), with a corresponding increase in the odds of exclusion (OR = 4.06; *p* < 0.0001). Moreover, one study examined the relation between socioeconomic indicators and listing outcomes through SIPAT domains: Perry et al. (2024) [21] found that patients insured through Medicaid had a threefold increased likelihood of exclusion for psychosocial reasons (OR = 3.24), predominantly mediated by the “readiness” and “social support” domains.

### Predictive validity of SIPAT for post-transplant adherence and acute rejection (*n* = 4 studies)

The predictive validity of the SIPAT has been primarily explored in relation to post-transplant medication adherence—often measured via the *Medication Level Variability Index* (MLVI)—and the incidence of acute rejection episodes. Deutsch-Link et al. (2021) [20] found that patients with SIPAT scores ≥ 21 had nearly threefold higher odds of post-transplant nonadherence (adjusted OR = 2.92), with the strongest associations observed in the readiness (aOR = 3.26), psychopathology (aOR = 1.88), and substance use (aOR = 3.03) domains. Supporting this, Zanatta et al. (2024) [24] reported that among 51 transplanted patients, those with SIPAT ≥ 21 were significantly more likely to exhibit suboptimal adherence (OR = 3.39; 95% CI: 1.07–10.26; *p* = 0.038).

Kim and Annunziato (2025) [23], using a matched analysis in a sample of 248 recipients, reported strong associations between “excellent” SIPAT Z-scores and favorable post-transplant outcomes, including low MLVI values and absence of acute rejection (*r* = 0.97–1.00). Conversely, “minimally acceptable” scores were associated with poorer clinical trajectories. However, Becker et al. (2021) [16], in a retrospective analysis of 182 patients, did not identify significant associations between total or domain-specific SIPAT scores and either MLVI > 2.5 or episodes of acute rejection, suggesting potential limitations of the tool in retrospective and categorical frameworks.

### Prediction of alcohol relapse risk (*n* = 3 studies)

Three studies evaluated relapse-related outcomes (Table 1). Dienstag et al. (2022) [14] compared 11 AAH recipients with 11 excluded candidates, reporting significantly lower SIPAT scores among those who underwent transplantation (23.3 vs. 49.5; *p* < 0.001). During a 6.6-year follow-up, 30% experienced harmful relapse, consistent with existing literature. Notably, the SIPAT proved effective for candidate selection even in the absence of the conventional “six-month abstinence rule.” In the same study, SIPAT scores were reported alongside other relapse-prediction tools (Michigan Alcoholism Prognosis Score (MAPS), High-Risk Alcoholism Relapse (HRAR), Alcohol Relapse Risk Assessment (ARRA), Hopkins Psychosocial Score (HPSS)), whereas Alcohol Use Disorders Identification Test–Consumption (AUDIT-C) and Sustained Alcohol Use post-Liver Transplant (SALT) did not significantly differentiate between groups [14]. Matthews et al. (2023) [17] found that SIPAT domains were significantly associated with relapse, despite no differences in mortality or rejection outcomes. Zanowski et al. (2022) [15]reported low sustained relapse rates (3.5%) in a cohort managed with SIPAT-informed selection and an integrated mental health approach, while emphasizing listing-related decision processes in severe alcoholic hepatitis.

### Evidence regarding quality of life outcomes (*n* = 0 studies)

Notably, none of the studies included in this review investigated the relation between SIPAT scores and post-transplant quality of life.

To facilitate comparison, we summarized key features of included studies in Table 1, providing a concise overview of designs, sample sizes, SIPAT scoring methods, and main outcomes.

**Table 1:** Summary of included studies evaluating the Stanford integrated psychosocial assessment for transplantation (SIPAT) in adult liver transplant candidates

Author (Year)	Ref	Country	*N*	Design	Patient status	Primary etiology	SIPAT version / language	Outcome domain	Key SIPAT findings
Sun et al. (2024)	21	USA	2762	Retrospective	Evaluated (listed vs. non-listed)	Mixed LT indications	Not reported (assumed standard version)	Listing	Higher SIPAT scores associated with non-listing; mediation by readiness and social support domains (SSDoH-related factors)
Deutsch-Link et al. (2021)	19	USA	1357	Retrospective	Evaluated / Transplanted	Mixed LT indications	Not reported (assumed standard version)	Listing; Adherence; Rejection	SIPAT ≥ 21 predicts non-listing and post-LT nonadherence; readiness domain associated with acute rejection
Perry et al. (2024)	22	USA	2825	Retrospective	Evaluated	Mixed LT indications	Not reported (assumed standard version)	Listing	SIPAT domains mediate socioeconomic disparities in listing decisions
Takano et al. (2023)	20	Japan	167 (71 LT)	Cross-sectional	Evaluated	Mixed (LT vs. other organs)	Japanese version	Distribution	LT candidates show higher SIPAT scores than heart/kidney candidates
Kim & Annunziato (2025)	23	USA	248	Cross-sectional	Transplanted	Mixed LT indications	Not reported (assumed standard version)	Adherence; Rejection	SIPAT Z-scores associated with MLVI and rejection; categorical cutoffs less informative
Zanatta et al. (2024)	18	Italy	134	Cross-sectional	Evaluated / Transplanted	ALD vs. non-ALD	Italian version	Distribution; Adherence	SIPAT ≥ 21 associated with poor adherence; higher scores in ALD
Zanowski et al. (2022)	15	USA	83	Prospective	Evaluated (listed vs. non-listed)	Severe AAH	Not reported (assumed standard version)	Listing; Relapse	SIPAT and social support predict non-listing; low sustained relapse with SIPAT-informed selection
Matthews et al. (2023)	17	USA	187	Retrospective	Evaluated	ALD vs. non-ALD	Not reported (assumed standard version)	Relapse	Higher SIPAT scores in ALD; domains associated with relapse but not mortality
Dienstag et al. (2022)	14	USA	22	Retrospective comparative	Transplanted vs. excluded	AAH	Not reported (assumed standard version)	Relapse	SIPAT differentiates eligibility and relapse risk; AUDIT-C/SALT not discriminative
Becker et al. (2021)	16	USA	182	Retrospective	Transplanted	Mixed LT indications	Not reported (assumed standard version)	Adherence; Rejection	No significant association between SIPAT and MLVI or rejection

Note. ALD = alcohol-associated liver disease; AAH = acute alcoholic hepatitis; LT = liver transplantation; MLVI = Medication Level Variability Index; SSDoH = structural social determinants of health

## Discussion

This scoping review critically examined the application of the *Stanford Integrated Psychosocial Assessment for Transplantation* (SIPAT) to adult liver transplant (LT) candidates, with three primary objectives: [1] to describe the distribution of total and subscale SIPAT scores among patients undergoing LT evaluation; [2] to explore differences in psychosocial profiles based on liver disease etiology; and [3] to assess the predictive validity of SIPAT in relation to listing decisions, post-transplant outcomes, relapse risk, and quality of life.

Overall, the literature indicates that a significant proportion of LT candidates fall within the moderate-to-high psychosocial risk range according to SIPAT classification. Mean SIPAT scores—often exceeding the threshold of 21 for clinically relevant risk [20, 24]—reflect the complex interplay of psychological, behavioral, and social factors interwoven with the medical condition of patients awaiting transplantation. Importantly, although ≥ 21 is frequently adopted in the included studies as a marker of elevated psychosocial risk, cut-off thresholds and risk categorization were not uniform across cohorts, which constrains direct cross-study comparability.

When situating SIPAT score distributions observed in liver transplant candidates within the broader solid organ transplant literature, prior cross-organ studies provide useful comparative context. In a mixed-organ candidate cohort from Japan, Takano et al. (2023) [22] reported higher total SIPAT scores among liver transplant candidates compared with heart transplant candidates, with differences particularly evident in psychosocial burden at the evaluation stage. Similarly, in a Thai validation study that included candidates for heart, liver, and kidney transplantation, Thisayakorn et al. (2021) [12] observed higher mean SIPAT scores in heart and liver candidates compared with kidney candidates, suggesting organ-related differences in psychosocial complexity. Consistent with these findings, the Spanish validation study by López-Lazcano et al. (2019) [7] reported that liver transplant candidates were more frequently classified into higher psychosocial risk categories compared with heart transplant candidates. Taken together, these cross-organ comparisons support the plausibility of relatively higher psychosocial risk profiles among liver transplant candidates in several settings, while also highlighting that absolute SIPAT distributions are highly sensitive to cohort composition, local listing practices, and health-system context.

The most critical domains are those related to treatment readiness, illness management, and substance use, suggesting that adherence challenges, limited illness awareness, and behavioral vulnerability constitute key elements of psychosocial risk in this population. However, domain-level reporting was inconsistent across the included studies, and the observation that “readiness” and “lifestyle/substance use” domains are most commonly impaired is primarily supported by the studies that explicitly reported domain patterns [24]; therefore, these domain-level inferences should be interpreted cautiously and not generalized beyond the available evidence base.

Observed differences among etiological subgroups—particularly between patients with ALD or AAH and those with other causes of liver failure—highlight SIPAT’s sensitivity in capturing specific vulnerability patterns. In the included evidence, etiological differences were directly assessed in a limited number of studies [17, 24]; therefore, etiological contrasts should be interpreted as mapped patterns rather than established general laws across LT settings. In ALD patients, higher SIPAT scores reflect not only a history of substance use, but also co-occurring psychological fragility, limited social support networks, and socioeconomic disadvantage [17, 21]. In this regard, SIPAT enables a nuanced characterization of psychosocial functioning by integrating clinical, relational, and contextual dimensions. This multidimensional approach is especially valuable in guiding individualized therapeutic strategies that move beyond rigid pre-transplant abstinence requirements toward structured motivational and rehabilitative pathways [14, 15].

A second key finding concerns SIPAT’s predictive validity for clinical and behavioral outcomes across both pre- and post-transplant phases. Several studies [19, 20] have demonstrated that higher SIPAT scores are independently associated with a lower likelihood of being listed for transplantation, indicating that psychosocial factors can significantly influence eligibility decisions. This association becomes particularly salient in the context of social disparities: patients insured through Medicaid or reporting low social support show markedly increased odds of exclusion on psychosocial grounds [21]. Here, SSDoH (structural social determinants of health) should be interpreted as structural dimensions of social inequality (e.g., insurance status and related socioeconomic constraints) that may shape access to transplantation through psychosocial evaluation processes. These findings suggest that SIPAT, beyond its diagnostic function at the individual level, may also act as a sensitive marker of structural inequities in transplant access, underscoring the importance of ethically grounded interpretation and equitable clinical application.

In the post-transplant setting, SIPAT has been consistently linked with medication nonadherence, variability in immunosuppressant drug levels, and—in some studies—episodes of acute rejection [20, 24]. The most predictive domains appear to be treatment readiness, psychiatric comorbidity, and substance use, reinforcing the value of pre-transplant psychosocial screening as a preventive measure against avoidable postoperative complications. In this sense, SIPAT functions not only as a diagnostic tool, but as a proactive clinical instrument capable of identifying high-risk individuals who may benefit from targeted psychosocial interventions and adherence support programs [18].

Although all eligible studies were included in accordance with scoping review methodology, the interpretation of findings in the present review was informed by study quality. Greater interpretative weight was assigned to patterns consistently supported by high-quality studies, particularly when similar associations emerged across independent cohorts and outcome domains. In contrast, findings derived from studies rated as moderate quality were interpreted more conservatively, primarily as signals of emerging associations or indicators of unresolved evidence gaps rather than as definitive evidence. This approach allowed us to balance the inclusive mapping purpose of a scoping review with the need to avoid overinterpretation in the presence of methodological heterogeneity.

Among its various applications, SIPAT’s ability to predict alcohol relapse risk stands out as particularly robust and clinically meaningful in the context of liver transplantation. Studies on ALD and AAH patients [14, 17] show that elevated SIPAT scores—particularly in domains assessing readiness, psychiatric comorbidity, and substance use—are significantly associated with a higher likelihood of harmful relapse post-LT, even in the absence of the conventional “six-month abstinence rule.” These findings support a paradigm shift toward a personalized, risk-based selection model focused on modifiable factors rather than fixed temporal criteria, thereby promoting more equitable and evidence-informed decision-making in transplant eligibility.

Despite the growing literature linking SIPAT scores to listing decisions, adherence, rejection, and relapse risk, a major gap identified by this scoping review is the complete absence of eligible studies evaluating associations between SIPAT scores and post-transplant quality of life outcomes. This finding is particularly relevant given the increasing clinical and policy emphasis on patient-reported outcomes, long-term functioning, and social reintegration after liver transplantation. Quality of life represents a core dimension of transplant success from the patient perspective, extending beyond graft survival and biomedical stability. Whether—and to what extent—pre-transplant psychosocial profiles captured by SIPAT domains predict post-transplant quality-of-life trajectories remains entirely unexplored within the current evidence base and should be considered a priority for future research.

From a clinical standpoint, one of SIPAT’s major strengths lies in its capacity to serve as a risk-informed rehabilitation tool. Rather than acting as a rigid exclusion criterion, it enables the identification of modifiable areas of vulnerability and the design of targeted psychological, educational, or social interventions. This approach aligns with the principles of a truly biopsychosocial model of transplantation, in which psychosocial evaluation is not limited to selection, but is integrated across the entire continuum of care [3].

Despite its strengths, several limitations warrant consideration. Most existing studies are single-center, retrospective, and methodologically heterogeneous, which limits generalizability and comparability. While SIPAT is increasingly used to structure psychosocial evaluation in liver transplantation, evidence regarding its internal structure is not fully consistent across studies. In particular, one included study raised concerns about structural validity and measurement invariance, suggesting that SIPAT domain scores and risk categorization may not operate uniformly across patient subgroups [21]. Moreover, inconsistent definitions of cutoff thresholds (e.g., ≥ 21 vs. ≥30) complicate the establishment of clinically meaningful benchmarks. Another underexplored area is SIPAT’s sensitivity to change—its capacity to detect improvements following psychosocial or rehabilitative interventions—which represents a crucial dimension for assessing its dynamic clinical utility. In addition, although SIPAT has been translated into multiple languages, our inclusion criteria were restricted to full-text articles available in English; therefore, relevant evidence published in other languages may have been missed, which should be acknowledged as a limitation of the present scoping review.

Future research should therefore prioritize prospective, multicenter studies aimed at validating standardized thresholds and evaluating SIPAT’s predictive power for long-term outcomes, including health-related quality of life, social reintegration, and graft survival. Refining the weighting of subscales could be explored to enhance its specificity for distinct clinical subgroups (e.g., ALD vs. MASLD), while integration with biological or behavioral adherence markers may further strengthen its multidimensional predictive capacity.

## Conclusions

This scoping review highlights the growing relevance of structured psychosocial evaluation of liver transplant candidates and identifies the *Stanford Integrated Psychosocial Assessment for Transplantation* (SIPAT) as a widely used instrument with potential clinical utility. Across heterogeneous studies, SIPAT scores were associated with listing decisions, post-transplant adherence, and relapse risk, although variability in study design, outcome definitions, and cutoff thresholds limits comparability and generalizability.

From a clinical perspective, SIPAT supports a shift from binary eligibility judgments toward a risk-informed, biopsychosocial framework focused on identifying modifiable vulnerabilities and guiding individualized care rather than serving as a stand-alone exclusion criterion.

Future research should prioritize prospective, multicenter studies to harmonize cutoff thresholds, assess sensitivity to change, and address the complete lack of evidence on post-transplant quality-of-life outcomes, a critical gap given the increasing emphasis on patient-centered and long-term transplant success.

## Data Availability

Not applicable.

## Abbreviations

AAH: Acute alcoholic hepatitis
ALD: Alcohol-associated liver disease
aHR: Adjusted hazard ratio
aOR: Adjusted odds ratio
ARRA: Alcohol Relapse Risk Assessment
AUDIT-C: Alcohol Use Disorders Identification Test–Consumption
CI: Confidence interval
HE: Hepatic encephalopathy
HPSS: Hopkins Psychosocial Score
HRAR: High-Risk Alcoholism Relapse
HRQoL: Health-related quality of life
IQR: Interquartile range
JBI-CAT: Joanna Briggs Institute Critical Appraisal Tools
LT: Liver transplantation
MAPS: Michigan Alcoholism Prognosis Score
MASLD: Metabolic dysfunction-associated steatotic liver disease
MELD: Model for End-Stage Liver Disease
MLVI: Medication Level Variability Index
OR: Odds ratio
PRISMA: Preferred Reporting Items for Systematic Reviews and Meta-Analyses
QoL: Quality of life
SALT: Sustained Alcohol Use post-Liver Transplant
SD: Standard deviation
SIPAT: Stanford Integrated Psychosocial Assessment for Transplantation
SSDoH: Structural social determinants of health
U.S.: United States
Z-score: Standardized score
